# Implant cleaning performance of powder abrasive devices: an in-vitro study on the non-surgical and surgical approach

**DOI:** 10.1186/s12903-026-08989-x

**Published:** 2026-07-04

**Authors:** Johannes Herzog, Alexandra Lüber, Milena Gemperle, Patrick R Schmidlin, Philipp Sahrmann

**Affiliations:** 1 Department of Periodontology, Endodontology and Cariology, University Centre for Dental Medicine, University of Basel, Mattenstrasse 40, Basel, 4058 Switzerland; 2 Clinic of Masticatory Disorders and Dental Biomaterials, Center of Dental Medicine, University of Zurich, Zurich, Switzerland; 3 Private Practice, Zurich, Switzerland; 4 Clinic of Conservative and Preventive Dentistry, Center of Dental Medicine, University of Zurich, Zurich, Switzerland

**Keywords:** Powder abrasive devise, Peri-implantitis, Biofilms, Minimal-invasive

## Abstract

**Introduction:**

To assess the cleaning potential of different powder abrasive devices (PADs) employing glycine powder in different peri-implant defect models, simulating the non-surgical and surgical approach.

**Methods:**

120 implants were stained with insoluble ink and mounted in models with circular steep and horizontal defects with and without simulated mucosa. Debridement was performed with three different commercially available PADs (1–3) for 120 s, either with a hand piece for the surgical or the nozzle for the non-surgical approach. Each treatment mode was performed on ten implants, respectively. Photos from each implant were taken under UV-A light activation and residually stained areas planimetrically assessed. Cleaning was tested for intergroup differences by two-way ANOVA with Bonferroni correction. Potential changes in the micro-morphology were investigated by scanning electron microscopy.

**Results:**

Taken all experiments together, device I performed significantly better than the others (*p* < 0.05). For the non-surgical approach, no significant overall difference was found. Horizontal defects were cleaned better than steep defects in all treatment-groups (*p* < 0.001). For the surgical approach, there was a significant overall difference between group I and III (*p* = 0.031). For both defect types, significant inter-group differences were found except for group II and III in the horizontal defect (*p* < 0.05). No configuration showed complete cleaning. Apically facing thread surfaces were found to be the most difficult to clean.

**Conclusion:**

(1) Different devices showed considerably different cleaning performances. Generally, stand-alone devices tended to perform better than powder chambers mounted on a handpiece. (2) Throughout the settings, steep defects are more difficult to clean.

## Summary Box

### What is known

In-vitro, powder abrasive devices provide a superior potential for simulated biofilm debridement. Different devices however, employing both the classic and the submucosal nozzle, have not been compared so far.

### What this study adds

Stand-alone devices seem to perform generally better in both defect types and, likewise, for simulated surgically and non-surgically performed cleaning. While steep defects showed more residual stains throughout the experiments, the three devices performed considerably different in the steep and horizontal defect types.

## Introduction

With a prevalence of 43% for peri-implant mucositis and 42% for peri-implantitis [1], inflammatory peri-implant diseases are considered a frequent disease [2] and the main reason for biological failures with dental implants [3]. The etiological reason for peri-implant diseases are oral bacteria which colonize on the non-shedding surfaces of implants and eventually develop into mature biofilm [4, 5]. Due to a subsequent inflammatory reaction of the host organism the peri-implant soft tissues display clinical symptoms like swelling, bleeding-on-probing and suppuration and the implant-supporting bone undergoes progressive destruction [6, 7].

Mechanical debridement is considered the adequate measure for biofilm debridement [8]. While being highly effective in areas with easy access to the respective surfaces, mechanical measures are limited in areas difficult to reach [9], and they might damage the surface morphology [10]. Especially titanium dioxide (TiO_2_), as a rather soft substance is prone to such changes of the surface structure [11].

In in-vitro studies, powder abrasive devices (PADs) with a low-abrasive powder like glycine have been shown to have a superior cleaning ability in typical defect configurations as compared to manual curettes or ultrasonic-driven devices [9, 12, 13]. Likewise, the least surface damage was noted by PAD with low-abrasive powders [9, 12, 13]. Today, there are many different devices for air abrasion on the market. These devices show different features like stand-alone or mobile powder chambers, parameters like powder ejection and water pressure, and spray angle, depending on whether a conventional handpiece is used or one for subgingival/submucosal usage. These differences are reflected in clinically relevant outcomes like differently homogeneous powder ejection, kinetic energy and finally the debridement potential of such devices.

Generally, biofilms on implants in the course of peri-implant therapy may be removed in a non-surgical (NSA) approach, or surgically (SA) after deflection of the peri-implant soft tissues and exposure of the affected surface areas. Often, a combination of both steps is performed as suggested in a recent guideline of the European Federation of Periodontology (EFP) [4].

While usually for SA conventional handpieces are used, providers suggest special tips. These so-called “nozzles” for subgingival or, in peri-implant therapy, submucosal use display a slim design, that allow for penetration of the pocket. In some cases, these nozzles cause also a change in the direction of powder emission which was shown to be beneficial for the cleaning of otherwise difficult-to-clean apically facing thread surfaces. For these nozzles different designs are available, that differ in terms of size, flexibility, and free rotatability on the handpiece.

Comparative tests between different systems under standardized application are largely missing.

Therefore, it was the aim of this in vitro study to compare the debridement efficacy of different powder abrasives devices with a low abrasive powder in typical defect morphologies for both the simulated non-surgical and surgical approach, with the null hypothesis that all devices would perform without significant differences in term of residuals stains.

## Materials and methods

### Defect models

**Fig. 1:**
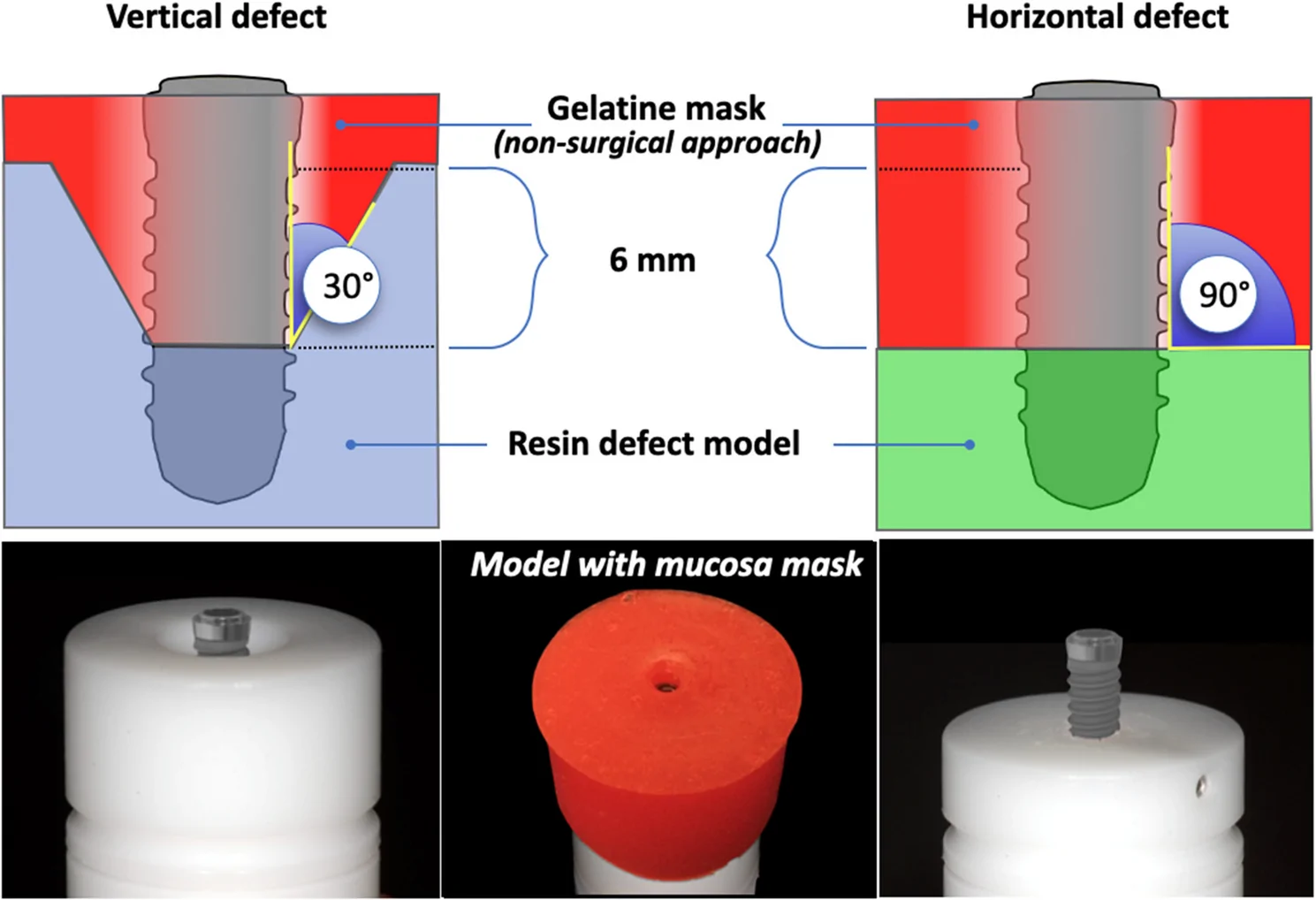
Defect models for the surgical and non-surgical approach

Defect models displaying circumferential 30° and 90° defects of 6 mm depth were made from polyoxymethylene (POM) (Polyoxymethylene - Copolymer; Merck, Zug, Switzerland). Implants were fixed in the model with a horizontal screw in a defined position, exposing 6 mm of the rough implant surface in addition to the machined collar (Fig. 1). The POM models displayed two circumferential grooves that allowed for exact positioning of a custom-casted artificial mucosa mask for NSA.

Before the experiments simulating NSA, 60 artificial mucosa masks were cast from red-stained and opaque (milk powder, Rapilait and Decora; Migros, Zürich, Switzerland) porcine gelatin (Ballistic 1; Gelita, Eberbach, Germany) as previously described [14], kept at -20 °C, and defrosted no more than 300 min before the mucosa masks were mounted.

The instrumentations simulating SA were performed on implants in defect models without mounted mucosa masks. Photos and schematic images of the defect models are given in Fig. 1.

Cylinder-shaped implants (SPI Element RC; Thommen Medical, Grenchen, Schweiz, length 11 mm, diameter 4.2 mm) with a machined neck (1.0 mm width, diameter 4.5 mm) and a roughness of 2.35 ± 0.25 μm for the intraosseous part were homogeneously stained with an insoluble high viscose varnish that is only visible if radiated with UV-A light (8280 Securitas UV Marker, Edding^®^; Thalwil, Switzerland) to simulate biofilm.

For each experiment, a virgin implant and – for NSA - a new mucosa mask used. For each setting (three devices, two defect morphologies, surgical or non-surgical approach) 10 implants each were tested. Further two implants were instrumented by each of the three devices without previous staining for SEM analysis. Accordingly, 260 implants were used for the entire experiment. More detailed information regarding the experimental setup is given in previous publications [12, 14].

### Instrumentation

**Fig. 2:**
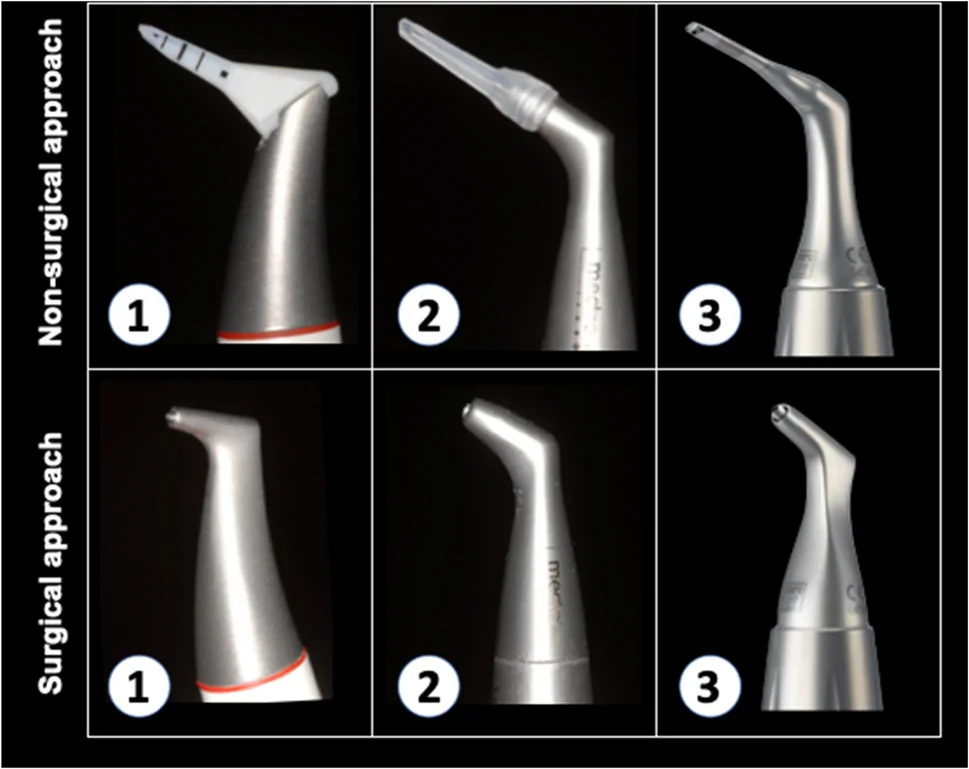
Device tips used for the simulated non-surgical and surgical approach in group 1, 2 and 3

According to the three different devices used the implants were divided into three groups according the device used (1 – EMS Airflow one; Electro Medical Systems SA, Nyon, Switzerland), 2 – Mectron combitouch; mectron spa, Carasco, Italy, 3 – Acteon Air’n’go easy; acteon, Colmar, France). Each device was equipped with their respective nozzle for subgingival/submucosal application for NSA and a normal hand piece for SA (see Fig. 2). The settings for water and power were set according to the manufacturer’s instructions (see Table 1). Throughout the entire experiment, glycine powder from the respective device manufacturers was used. These powders were, however, identical and produced by the same company (3 M Espe, Seefeld, Germany). While for group 1 and 2 the single-use nozzles were used only once, the steel nozzle for the device in group 3 was flushed between the single experiments but not changed. Treatment time for each implant was exactly 120 s.

**Table 1:** Device settings

Group/device	Device	Power and flushing settings	Simulated Approach		Glycine Powder
			Non-surgical	Surgical	
1	EMS AIR-FLOW^®^ ONE (Stand-alone) *(EMS*,* Nyon*,* Switzerland)*	powder: 10/10, water 1*	Perioflow handpiece with subgingival nozzle (single use)	Conventional handpiece	AIRFLOW PERIO Powder 25 μm
2	Mectron combi touch (Stand-alone) *(Mectron*,* Carasco*,* Italy)*	water 10/10*	Sprayhead perio with subgingival tips (single use)	Normal hand piece	Mectron glycine Powder 25 μm
3	AIR-N-Go^®^ Easy Turbine clutch at 3 bar air pressure *(Acteon*,* Merignac*,* France)*	(no adjustable settings)	AIR’N’GO^®^ Perio nozzle for multiple use	Normal hand piece	AIR N GO Perio powder 25 μm

* Powder and water settings as indicated by the manufacturer as optimal settings ®-registered trademark

All instrumentations were performed by two operators (AL and MG), each working on one half of the samples. Treatment chronology and allocation of the different devices was performed according to a previously generated computer random list (www.random.org).

### Assessment of residual stains

**Fig. 3:**
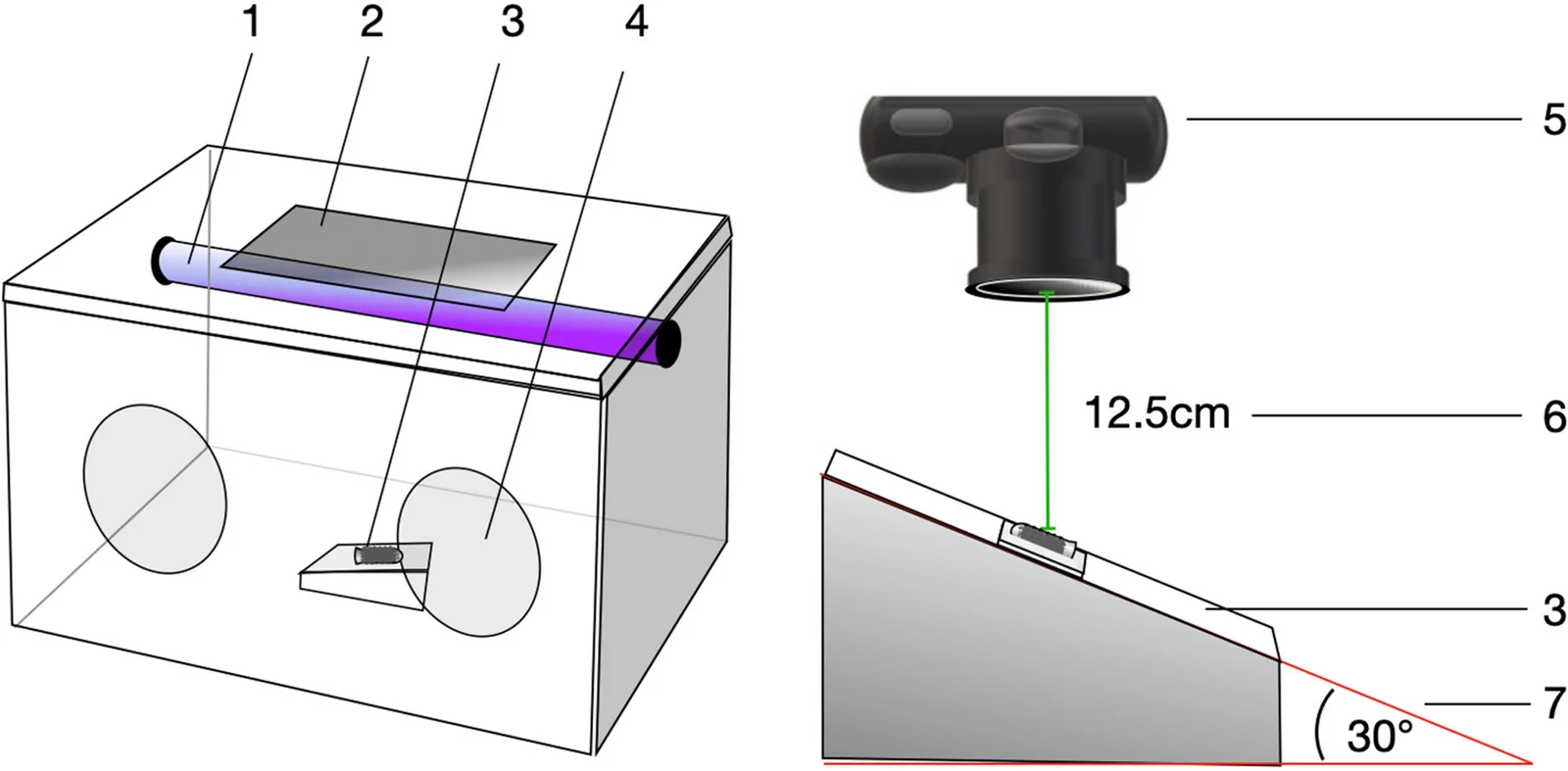
Settings the assessment of residual stains

After the 120 s of treatment, implants and mucosa masks (for the surgical approach) were taken off from the model. Loose stain particles were removed under gentle water flow and the implant was dried by airflow. Photographs were taken through the transparent window of an otherwise lightproof box (dimensions 386 × 215 × 285 mm, (L ×H ×W)) with UV-A illumination (Didaktobox™; Mundipharma Medical Company, Basel, Switzerland, illumination at 365 nm, 8 W). UV-A was used to induce fluorescence from the residual stains on the implant surfaces, rendering these particles well visible. Within the box, implants were placed on a stand, placing the implant axis in an angle of 60° to the direction from where the photo was taken (see Fig. 3). Thus, pictures were taken slightly from apically. Photos were taken under standardized conditions (D200; Nikon, Tokio, Japan with photo lens Sigma 105 mm; Sigma, Rödermark, Germany, shutter priority at 1.0 s exposure, distance to implant 12.5 cm). A blinded examiner, unaware of the group allocation of the respective implants, trimmed the photos back to the area of interest, converted them into binary images (Keynote; Apple Inc, CA, USA, Vs. 17.2) and performed the analysis of the residually stained surface using a custom-made planimetric application (Image 1.61 FAT; PPK, ZZMK, Zurich, Switzerland). In a sub-assessment for different surface areas (machined neck (MN), thread tips (TT), area between the tips (BT) and apically facing thread surfaces (AT), residual staining was classified according to a score indicating the cleanliness of the respective segment as follows: 6–95%, 5–95 − 75%, 4–75 − 50%, 3–50 − 25%. 2–25-5%, 1 - <5% of residually stained surface [12]. This was done to detect the most difficult-to-clean areas for each approach and defect (Table 2). Scoring was performed by two independent investigators (AL, MG) and the mean of their score was used for further analysis. 

**Table 2:** Uncleaned surfaces per group and defect and treatment approach

	Defect type	1	2	3
Non-surgical approach	Horiz. (90°)	10.89 ± 6.34 ^A^	18.08 ± 8.44 ^BC^	18.21 ± 8.18 ^BC^
	Vert. (30°)	28.15 ± 6.86 ^A^	32.82 ± 12.85 ^A^	28.19 ± 6.11 ^A^
Surgical approach	Horiz. (90°)	3.19 ± 1.11 ^A^	8.90 ± 5.64 ^BC^	10.23 ± 3.03 ^BC^
	Vert. (30°)	28.12 ± 5.41 ^A^	32.40 ± 3.36 ^B^	44.12 ± 9.41 ^C^

Different superscripts with same line indicate significant differences (t-test, *p *< 0.05)

### Assessment of surface morphology changes

Scanning electron microscopy imaging (Carl Zeiss Supra 50 VP FESEM; Carl Zeiss, Oberkochen, Germany at 10 kV, working distance 9 mm) was performed on the instrumented machined and rough surface areas to check for possible changes in the surface morphology. This assessment was performed under 5,000 and 10,000-fold magnification, comparing these images to respective surface areas of untreated control implants.

### Statistics

A sample size calculation was conducted, suggesting a main difference of 10 ± 10% for the means and standard deviation of the cleaned surface proportion. Given a power of 80% and a level of significance of 0.05 a minimal sample size of 37 was calculated. Thus, 40 implants per device were set as sufficient sample size. For each group, means, standard deviations (SD) and interquartile ranges (IQR) of residually stained were calculated. Given a positive test on normal distribution of the standard deviations, differences for the respective devices, defect angulations and surgical or non-surgical approaches were tested using parametric two-way ANOVA for unpaired datasets. The level of significance was set at *p* < 0.05. All statistics were calculated with SPSS Statistics^®^ Version 23.0.0.2; IBM, SPSS, IL, USA).

No ethics approval was needed since no patients or human tissues had been used for the present study.

## Results

### Overall intergroup comparison

For both, the surgical and non-surgical approach taken together for both defect types, significant differences (*p* < 0.05) were observed between group 1 and the other groups, displayed by residually uncleaned surface proportions of 17.59 ± 12.20%, 23.05 ± 13.0% and 25.20 ± 14.53% for group 1, 2, and 3, respectively.

### Non-surgical approach

In the overall analysis of residually stained surfaces in all defects, there was no significant inter-group difference regarding residual stain, with a residually stained surface proportion of 19.52 ± 10.95%, 25.45 ± 13.00%, and 23.20 ± 8,67% for group 1, 2 and 3, respectively (means ± std). Generally, the horizontal defect was found to be cleaner than the steep defect with residually stained surfaces of 15.731 ± 8.21% and 29.72 ± 9.08%, respectively (*p* < 0.001). A sub-analysis for the different devices revealed that this difference was also detectable in each single group (see Table 2).

**Fig. 4:**
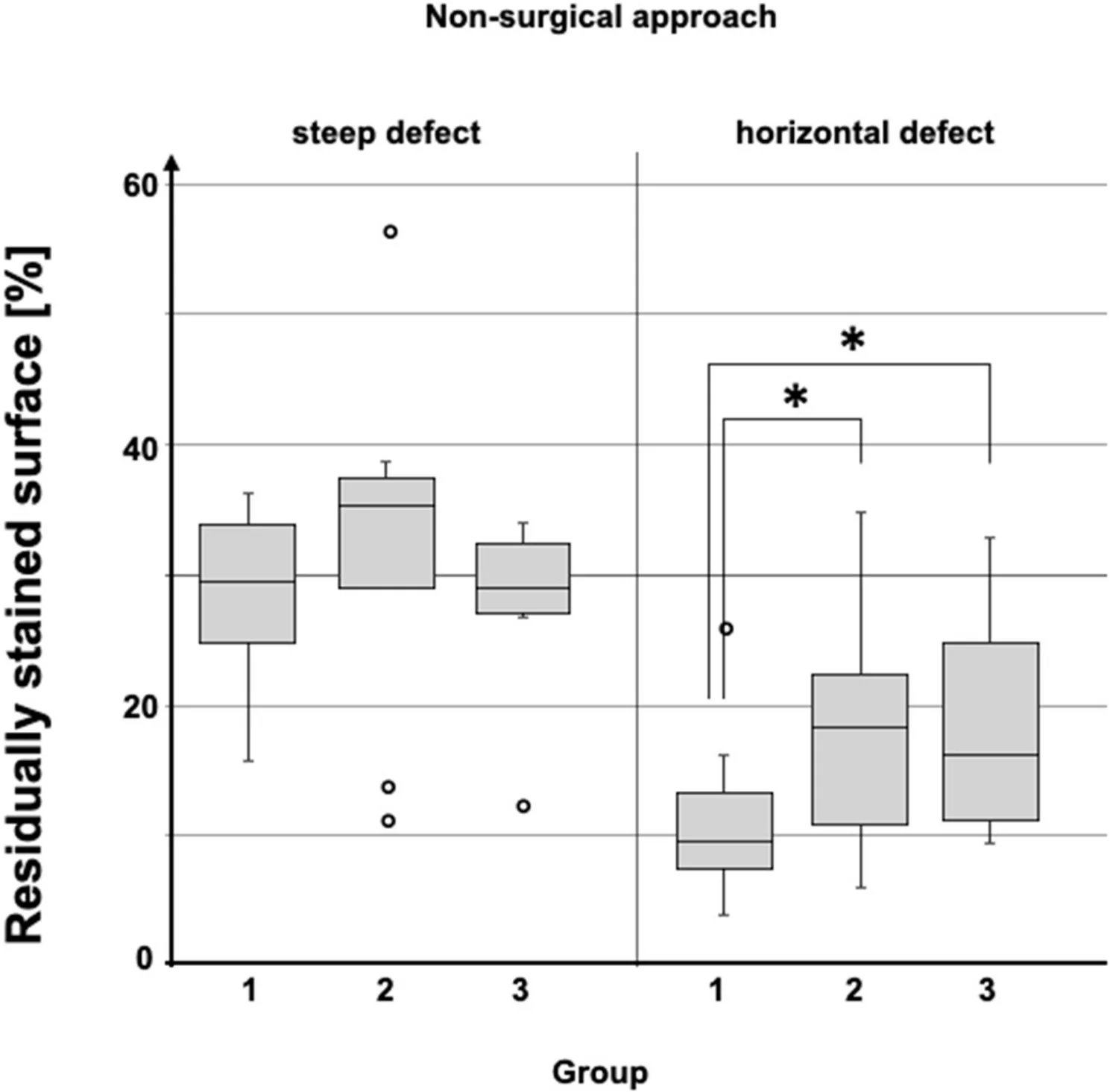
Residually stained surfaces (non-surgical approach)

While in horizontal defects device 1 performed significantly better than the other devices, no such differences were found in the vertical defects, as indicated in Table 2; Fig. 4.

A complete removal of simulated biofilm was found in none of the defects for any of the groups.

### Debridement scores

**Fig. 5:**
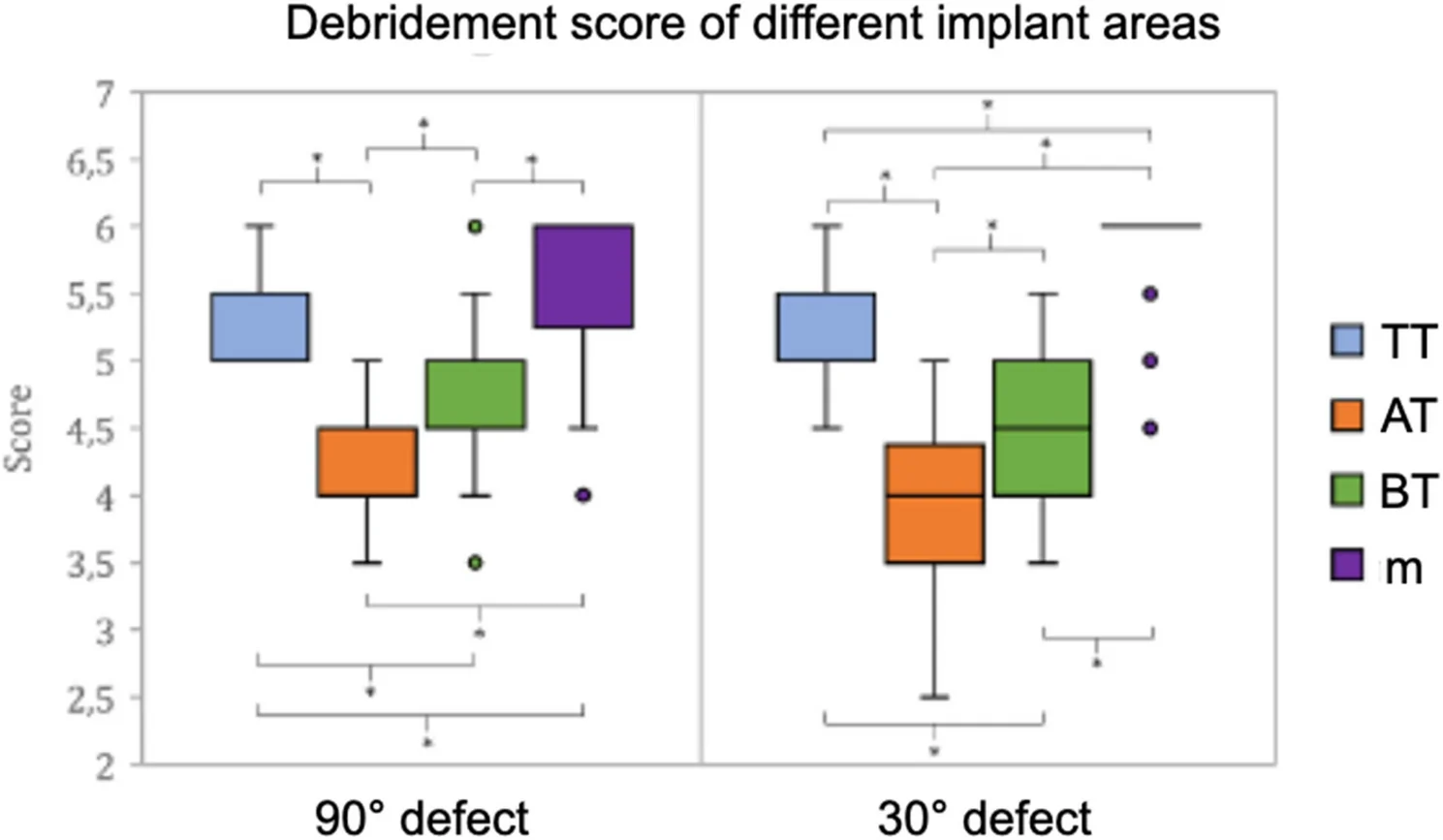
Debridement scores for the steep and horizontal defects. **a **Non-surgical approach (**b**) Surgical approach

Assessing different surface areas by a debridement score, for both defect types the lower thread surfaces displayed the most residuals followed by the area between the threads, while the area with the least residuals was the machined surface area at the implants neck, followed by the tip of the threads. Regarding the apically facing thread areas and the areas between the threads in the difficult-to-clean steep 30° defect, there were significant inter-group differences, favoring group 1 before the others, and group 3 before group 2 (*p* < 0.05) (see Table 3 and Fig. 5).

**Table 3:** Results for the debridement scores. Non-surgical approach

Device	90° defect			30° defect		
	1	2	3	1	2	3
Edge of thread (ET)	5.15 ± 0.24	5.25 ± 0.35	5.3 ± 0,35	5.0 ± 0.24	5.3 ± 0.35	5.25 ± 0.51
Lower thread [15]	4.35 ± 0.53	4.25 ± 0.49	4.55 ± 0.28	4.45 ± 0,60	3.35 ± 0.47	3.75 ± 0.49
Between threads (BT)	4.9 ± 0,46	4.85 ± 0.63	4.6 ± 0,61	5.05 ± 0.37	3.9 ± 0.39	4.45 ± 0.44
Machined surface (MS)	5.65 ± 0.58	5.45 ± 0.90	5.8 ± 0.42	5.7 ± 0.54	5.8 ± 0.48	6 ± 0

Score 1-6 refer to the following percentage of cleaned surface area: 6 – 95%, 5 – 95-75%, 4 – 75-50%, 3 – 50-25%. 2 - 25-5%, 1 - <5%

### SEM assessment

In none of the groups any changes of the surface morphology were detectible in terms of either scratches on the machined or rough surface area, or residuals from powder or the resin nozzles.

### Surgical approach

**Fig. 6:**
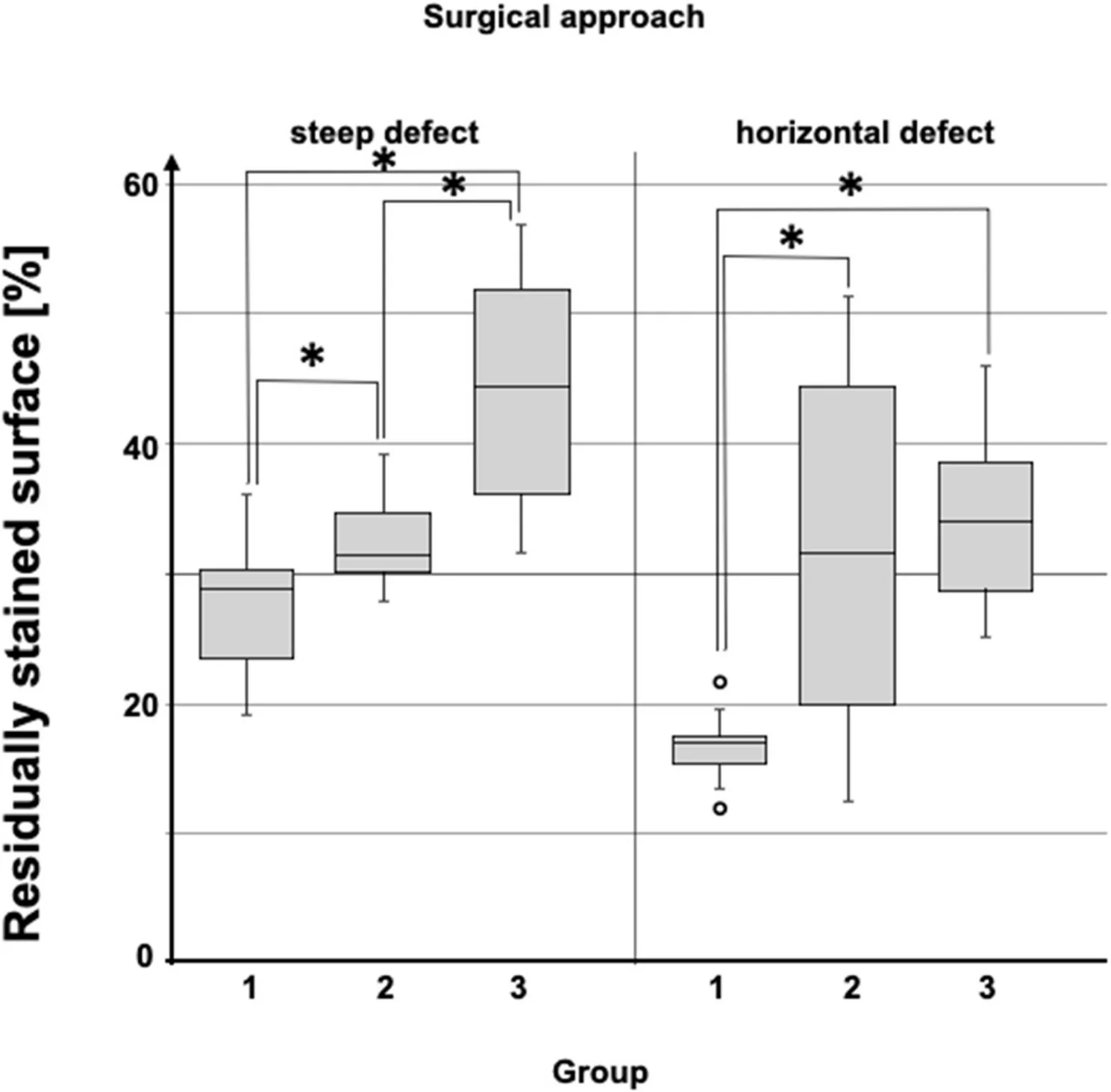
Residually stained surfaces (surgical approach)

The overall analysis of residually stained surfaces in all defects revealed values of 15.66 ± 13.35%, 20.65 ± 12.88%, and 73.20 ± 18,69%, showing a significant difference only between group I and III (*p* = 0.031). All groups taken together, implants with a horizontal defect were cleaned significantly better than those with a vertical defect, with residually stained surfaces of 7.43 ± 9.36% and 34.90 ± 9.36%, respectively (*p* < 0.001). A sub-analysis for the different devices revealed that this was also true in each single group (see Table 2; Fig. 6).

In both, the horizontal and the vertical defect, statistically significant inter-group differences were found, except for the horizontal defect in group II and III (*p* = 0.520).

A complete cleaning was not found in any of the defects in any of the groups.

### Debridement scores

**Fig. 7:**
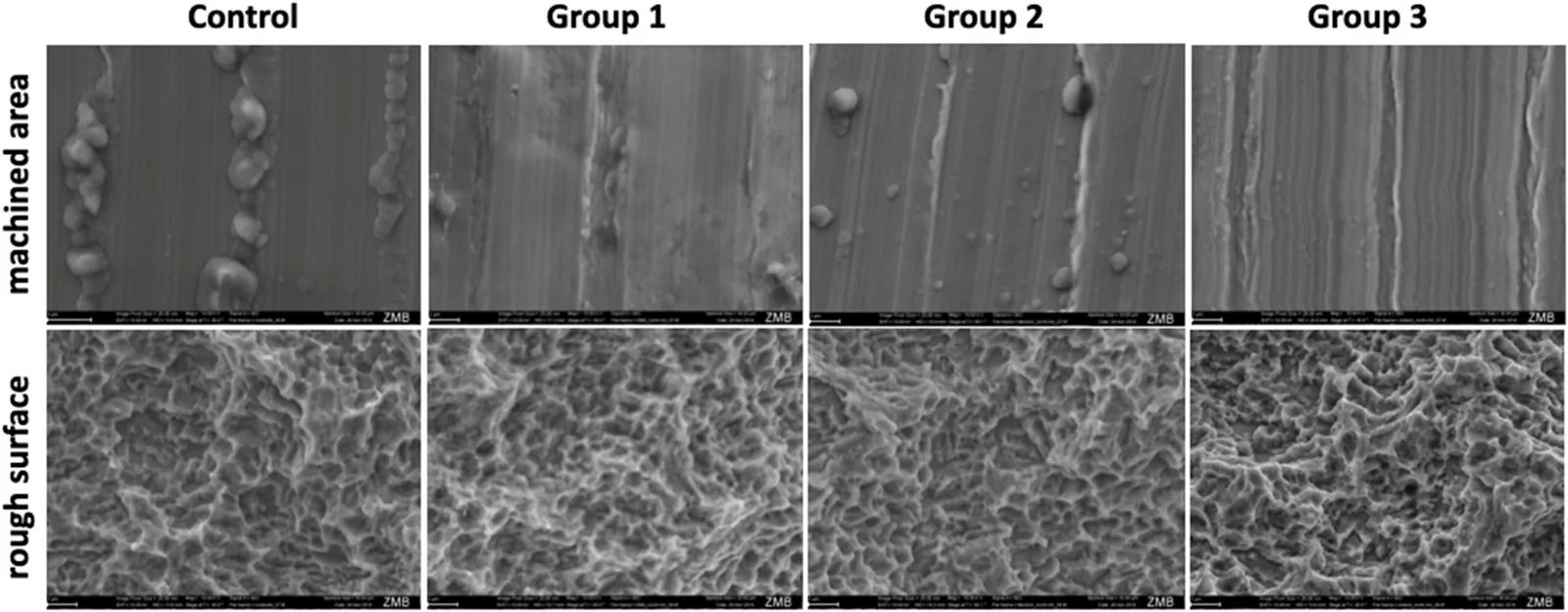
SEM images of the machined and rough surface areas after treatment with glycine powder abrasion for 120 s with devices 1, 2, and 3 at 10’000-fold magnification

In the surgical approach, different surface areas showed significantly different results when the debridement score was applied. In both defect types the lower thread surfaces displayed the worst result. In the steep defect, the area between the threads showed the second most residually stained surface proportion, while the machined part, the area between the threads in the horizontal defect and the tips of the threats were cleaned rather well (Table 4, Fig. 7).

**Table 4:** Results for the debridement scores. Surgical approach

Device	90° defect			30° defect		
	1	2	3	1	2	3
Edge of thread (ET)	5.00 ± 0.0	5.00 ± 0.0	5.00 ± 0.0	6.00 ± 0.0	5.2 ± 0.42	5.10 ± 0.32
Lower thread [15]	3.00 ± 0.0	2.00 ± 0.0	2.00 ± 0.0	5.3 ± 0.48	2.5 ± 1.18	3.5 ± 0.53
Between threads (BT)	2.00 ± 0.0	2.60 ± 0.52	3.00 ± 0.0	6.00 ± 0.0	6.00 ± 0.0	6.00 ± 0.0
Machined surface (MS)	6.00 ± 0.0	6.00 ± 0.0	6.00 ± 0.0	6.00 ± 0.0	6.00 ± 0.0	6.00 ± 0.0

Score 1-6 refer to the following percentage of cleaned surface area: 6 – 95%, 5 – 95-75%, 4 – 75-50%, 3 – 50-25%. 2 - 25-5%, 1 - <5%

Again, in the difficult-to-clean steep 30° defect, the problematic apically facing thread areas were cleaned significantly better by the device in group 1, followed by group 3 (*p* < 0.05) (see Table 4).

### SEM imaging

Surfaces did not show any optical changes, neither on the machined nor on the rough surface area of the implants, after instrumentation by any of the three devices (see Fig. 7).

## Discussion

The present in-vitro study comprehensively assesses the cleaning performance of different powder abrasive devices in two peri-implant defect morphologies as accessed by simulated non-surgical and surgical means.

Generally, steep defects were cleaned less completely while a complete cleaning was found in none of the defects. While there was no significant inter-group difference in the overall cleaning capacity, in the steep defect configurations better results were obtained with the device used in group 1. This was the case for both, the surgical and the non-surgical approach. Therefore, the null-hypothesis had to be rejected.

Generally, machined surfaces in the shoulder areas were cleaned well, while apically facing thread surfaces turned out to be the most difficult to clean. These results corroborate with a previous analysis performed on screw-formed implants with moderate rough surface (Straumann wide neck tapered effect implants with SLA surface (non-surgical approach) and Thommen SPI Element RC implants with Inicell^®^ surface for the surgical approach) [12].

The superiority for stand-alone devices might be explained by a more continuous and homogeneous powder flow, when the powder chamber remains in a fixed position instead of being turned over [16]. Regarding the different performance of the stand-alone devices, however, the design of the nozzle and the respective direction of powder ejection might be reasonable explanations: While device 1 claims to eject powder in three directions in a vertical plane to the tooth axis, device 2 is meant to eject the powder in only two directions. For device 3 ejection results in two directions in a bold angle to the tooth axis. In the latter case, debridement of the apical facing threads is difficult to imagine.

In a recent in-vitro study on the cleaning performance of powder abrasive devices on horizontal defects around apically or completely tapered implants, a proportion of 45 − 25% of residually stained surface was found, employing the device used in group 1 in the present study [17]. The considerably worse cleaning in that study may be attributed to several differences in the in-vitro set up, namely a different color for plaque simulation, stiffer peri-implant mucosa and a considerably deeper defect of 10 mm. Likewise, the implants used (Straumann BLX implants) were described as displaying an “aggressive” thread design which supposedly displays surface areas that are more difficult to access [17]. Previous studies, however, show comparable results to the present study for both, the vertical and horizontal defects [9, 14, 18]. Important to note, that in the present study another ink was used, which was detectable only by black light activation. This fact suggests an indirect proof of validity for the use of the novel, normally invisible ink [9, 14, 18]. In another in-vitro study simulating the surgical approach with a similar set-up but employing hydroxyapatite to simulate biofilm, a group from Tokyo assessed the cleaning performance in the coronal, middle and apical third of a 3 mm deep moderately steep defect, finding a mean value of 15.4 ± 2.8% of uncleaned surface areas, with a trend for higher values in the deeper areas of the defect [19]. Wei et al. [20] assessed the effect of different air pressure on the efficacy of air abrasive devices. Employing glycine at a pressure of 35–55 psi, i.e. 2.4–3.8 bar, similar cleaning results to the present study (in which similar pressures were used) were found, while a pressure of 25 psi (1.7 bar) resulted in considerably inferior result.

While powder abrasion with glycine has consistently shown promising results in vitro, the clinical benefit of its use is still matter of ongoing discussion: In a systematic review Schwarz et al. concluded, that while there does not seem to be a benefit as compared to conventional cleaning methods like ultrasonic scalers with carbon [21] or resin tips [22] or Teflon [23] in sites with peri-implant mucositis, its non-surgical use in the diseased peri-implant site might be of benefit [24]. In the recent EFP guideline for peri-implantitis treatment, however, its use is not recommended, due to the fact that the acquisition is rather cost intense and there is a lack of clinical data from randomized clinical trials that would report superior effects in comparison to hand or ultrasonic scaling [15]. While it is difficult to point out with certainty the reason for the mismatch of results from in-vitro and clinical studies it is likely, that the study duration of 3–6 month [25, 26] after a single treatment of implant debridement measures results in equally modest results for all treatment groups not due to the cleaning efficacy of the different devices used but due to the quick regrowth of submucosal biofilm during the considerable follow-up period of several months [27–29].

The present in-vitro assessment suffers from two major limitations: First, the debridement capacity was not tested on a true oral biofilm for several reasons: Ex-vivo biofilm may differ considerable between test persons and their way of life, laboratory biofilms do not depict the mechanical characteristics of true oral biofilm and is prone to be removed already by waterjet, what is not true for true oral biofilms [30]. Furthermore, quantitative assessment of biofilm remnants by assessing regrowth of bacteria by counting colony forming units is a hardly reliable method due to the indirect and complex analysis. Instead, for the present experiments, an insoluble, waterproof ink was used. This has been done for the advantage of a highly standardized simulation in terms of thickness, composition and homogeneity. While this ink was not removable by mere waterjet but only by powder, it has not yet been tested, whether the removal of true biofilm is comparable to that of the ink used. Since it is reasonable to suggest a more difficult removal of ink than biofilm, the simulation is considered less sensitive on removal, but likewise less prone for false-negative results. It constitutes, however, due to its easy and standardized way of application, a valid way to test for inter-group differences if different devices are tested and compared. Then, the simulation of osseous defects and – for the non-surgical approach – peri-implant mucosa comes along with immanent limitations. While bleeding of probing as a factor that might hamper the controlled debridement process could not be simulated, the more complex and inestimable defect morphology of a true peri-implant defect might not be fully reflected by the model. On the other hand, the present simulation offers a well-standardized method to compare debridement efficacy of different powder abrasive devices. While the present manuscript describes a comprehensive design involving three different devices in both, steep and horizontal, defects and addressing the non-surgical and surgical approach.

The use of an ink that is invisible to the naked eye is a novel approach in the assessment of debridement on dental implants. Results comparable to those of previous experiments and with a relatively low deviation suggest a good applicability of this kind of plaque simulation.

Since the present type of biofilm simulation has been adapted by various studies, future studies should assess the comparability of insoluble ink and ex-vivo biofilms [30]. This is needed to validate the present results and might potentially help to enhance the characteristics of the biofilm-imitating substance [31].

## Conclusions

Within the stated limitation of the present study design it seems that there are, in fact, differences in the debridement efficacy of different powder abrasive devices during both the non-surgical and surgical approach. Thereby, stand-alone devices tended to perform better than powder chambers mounted on a handpiece. Generally, steep defects prove to be more difficult to clean than horizontal defects, even if nozzles for submucosa use were employed.

## Data Availability

Raw data may be provided upon reasonable request by the corresponding author.
